# Study on the contrast of the MHC–peptide interaction of B2/B21 haplotype and MHC‐related virus resistance in chickens

**DOI:** 10.1002/iid3.520

**Published:** 2021-09-02

**Authors:** Yuan‐chang Jin, Wei Wang, Min‐min Yu, Mei‐lin Hao, Gang Zeng, Jing‐fen Chen, Juan Dai, Yu‐jie Wu

**Affiliations:** ^1^ College of Biology and Agriculture (College of Food Science and Technology) Zunyi Normal College Zunyi Guizhou China; ^2^ School of Life Science Hunan University of Science and Technology Xiangtan Hunan China

**Keywords:** antigen presentation, chicken, MHC I‐related molecules, virus resistance

## Abstract

**Introduction:**

Three‐dimensional (3D) structures of MHC class I exert some influence by the MHC–peptide interaction over host resistance to the virus. The thesis aims at studying the connection between MHC–peptide interaction of B2/B21 haplotype and MHC‐related resistance to the virus.

**Methods:**

The structure of chicken MHC class I BF2*0201 from B2 haplotype was studied and contrasted with that of BF2*2101 from B21 haplotype by using DNAMAN and PyMol software.

**Results:**

The amino acid difference resulted in the difference in size and changeability of the binding groove of the two, resulting in different choices on the binding polypeptide. 3bew's (the crystal structure of BF2*2101 bound to peptide RV10) small side chain His111 replaces the short side chain Tyr111 of 4cvx (the crystal structure of BF2*0201 bound to peptide YL9), and the very small amino acid of Ser69 and Ser97 make the middle of the 3bew's binding groove become apparently broad and bound restrictive of amino acid smaller. Moreover, due to the specific amino acids—Arg9, Asp24, and Asp73 of 4cvx and Arg9, Asp24, and His111 of 3bew, the effect of the polypeptide and the binding groove differ between the two, and 3bew tends to bind polypeptides with negatively charged amino acids, but the large space in the middle can also accommodate other amino acids. Contrasted with the binding groove characteristic of 4cvx, it can be said that the selectivity of 3bew is higher than that of 4cvx in the amino acid type of the binding polypeptide, so the B21 haplotype has more host resistance to the virus than that of the B2 haplotype in chicken.

**Conclusion:**

There are usually various kinds of peptides presented by the BF2*2101 molecules of B21 haplotypes, resulting in resistance to pathogenic microorganisms, such as Rous sarcoma virus and/or Marek's disease virus. These findings may have an important theoretical foundation for screening of virus antigen, vaccine design, and genetic resistance breeding.

## Graphical abstract

The research is dedicated to the 3D structure of 4cvx from the B2 haplotype and the contrast of the BF‐peptide interaction of 4cvx and 3bew from the B21 haplotype. Conclusion of this study: There are usually various kinds of peptides presented by the BF21*2101 molecules of B21 haplotypes, resulting in resistance to pathogenic microorganisms, such as Rous sarcoma virus (RSV) and/or Marek's disease virus (MDV).

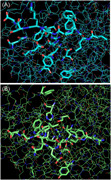

## 1 INTRODUCTION

As one of the chief functions of MHC class I, endogenous presentation of derived peptides to T lymphocytes is of coercion in numerous pathological conditions. The influence on levels of MHC class I give rise to immune escape of pathogens or cancer cells. It is consequently significant to recognize the molecular mechanisms of expression, processing, and antigen‐presenting of MHC class I.^1^, ^2^

As central in defining the responses' character of adaptive immune, MHC class I provide a context in that pathogenic microorganisms are recognized. The BF genes (MHC class I genes of chicken) involve two classes of Ia genes: BF1 and BF2 genes.^3^ The major step forward in defining the three‐dimensional (3D) structure of the BF2 and in demonstrating its function was made. It is exactly the BF2 molecules that take charge of classical peptide antigen‐presenting.^4^, ^5^ BF2 of susceptible haplotypes can bind only one or a few like peptides, with stringent peptide motifs and narrow peptide repertoires,^6^ while the BF2 of resistant haplotypes a greatly most category of amino acids and with relaxed peptide motifs and wide peptide repertoires.^7^

Until now, the structural and functional researches about the BF2 molecules are limited. The aim of the study was to carry out extensive research into the 3D structure of BF2*0201 from the B2 haplotype by PyMol and DNAMAN software, and contrast it with the known 3D structures of BF2*2101 from the B21 haplotype to clarify the architectural feature of BF2*0201 binding antigenic peptides and further to research the connection between the interaction of BF2‐peptide and BF2‐related pathogen resistance.

## 2 MATERIALS AND METHODS

### 2.1 Amino acid sequences differences between 3bew and 4cvx

From Protein Data Bank (PDB) website (https://www.rcsb.org/), the fasta files of 3bew (the crystal structure of BF2*2101 bound to peptide RV10) and 4cvx (the crystal structure of BF2*0201 bound to peptide YL9) amino acid sequences were obtained, both including A, B, and C chain. For the 3bew, α1–α2 super domain of the A chain was to be contrasted with that of 4cvx by using DNAMAN software.

### 2.2 DSSP differences between 3bew and 4cvx

From the PDB website, the definition of the secondary structure of proteins (DSSPs) of 3bew and 4cvx were obtained, respectively. It was analyzed the DSSP differences between 3bew and 4cvx on A, B, and C chains.

### 2.3 Carbon backbone differences between 3bew and 4cvx

The overall carbon main chain of 3bew and 4cvx were analyzed, respectively, and then contrasted, and the RMSD value of the carbon main chain was contrasted from the internal molecular.

### 2.4 Peptide binding grooves differences between 3bew and 4cvx

By using the PyMOL molecular graphics system (DeLano Scientific, http://www.pymol.org), electrostatic potential surfaces and structural figures of 3bew and 4cvx were generated, respectively, and under the same magnification, the peptide‐binding grooves of 3bew and 4cvx were contrasted visually with one another. The amino acids constituting the peptide‐binding grooves were found by using PyMol software. The amino acid residues that consist of each pocket of the binding grooves were extracted to analyze the differences of the hydrophilic/hydrophobic and charge properties of the pocket as well as the specific amino acids of peptide binding grooves between 3bew and 4cvx.

### 2.5 Differences in the interaction of peptide and binding grooves between 3bew and 4cvx

By using PyMol software, to explore and speculate on the peptide presentation rules of BF2 molecule, the differences in the interaction of peptide and binding grooves between 3bew and 4cvx were carried out.

## 3 RESULTS

### 3.1 The contrast of overall structure of BF2*0201 and BF2*2101

**Figure 1:**
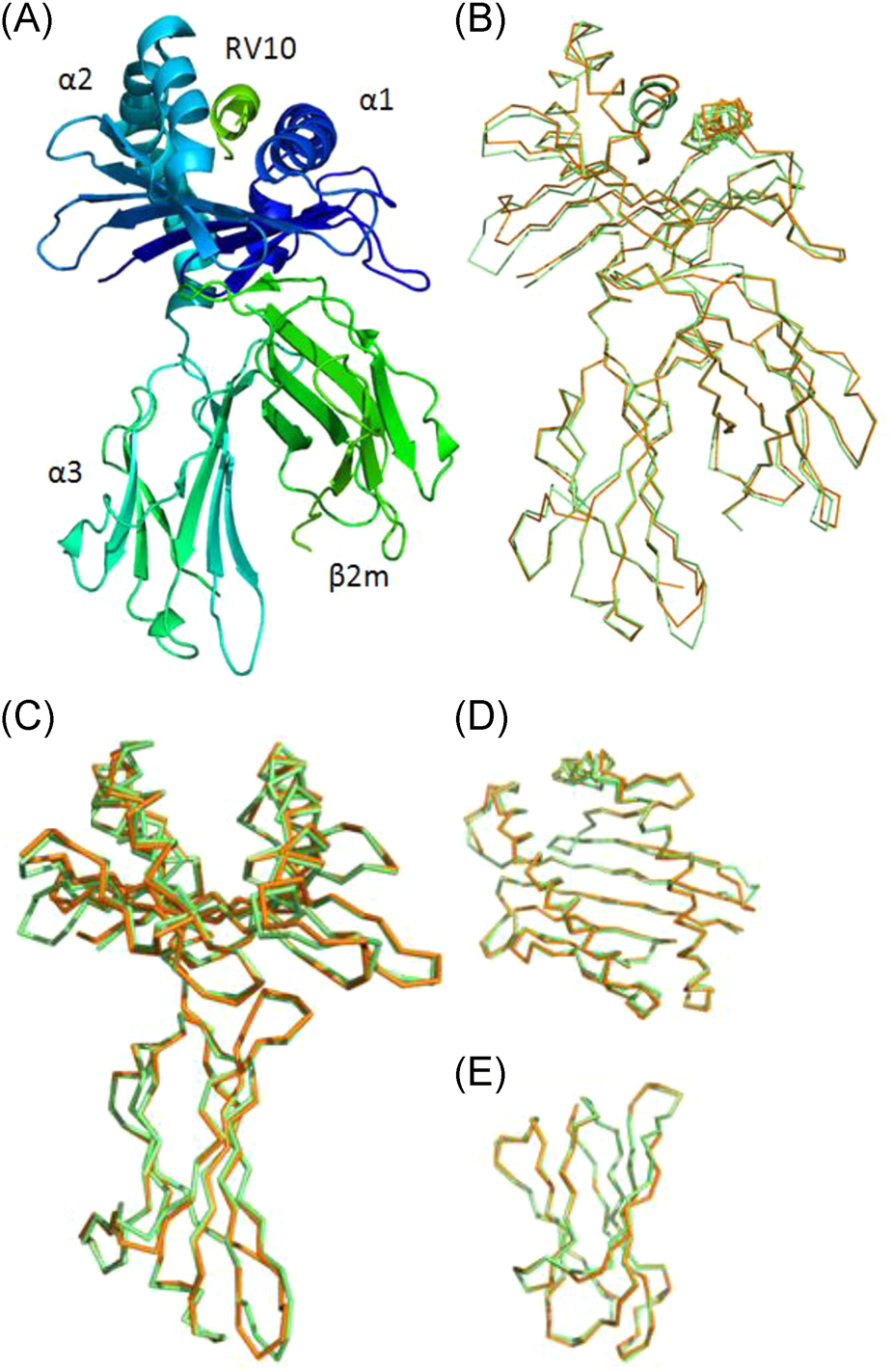
The structure of the chicken MHC I molecule BF2*2101 (PDB code: 3bew) and its comparison with BF2*0201 (PDB code: 4cvx). (A) The overall structure of chicken BF2*2101, a typical MHCI structure. (B) Superimposed Cα‐traces of chicken BF2*0201‐YL9 (orange), BF2*2101‐RV10 (green). It shows that the overall structures of the two chickens MHC I are very similar. (C–E) Carbon backbone deviation analysis of BF2*0201‐YL9 and BF2*2101‐RV10. (C) The ratio of the carbon backbone of the A chain. (D) The ratio of the carbon backbone of the α1–α2 chain. (E) The ratio of the carbon backbone of the B chain

The crystal structure of BF2*0201 (PDB code: 4cvx) bound to peptide YL9 (YPYLGPNTL) was solved by molecular replacement using the BF2*2101 structure (PDB code: 3bew) bound to peptide RV10 (REVDEQLLSV) as a search model. In brief, the α1 and α2 domains make up the binding groove, with two α‐helices atop an eight‐stranded β sheet. The α3 domain resembles that of chβ2m, both consisting of two sets of opposite and parallel β sheets. The α1/α2 and α3 were integrated by noncovalent bonds. In a position to interact with the receptor of T cells, the RV10 peptide binds to the top groove of the 3bew (Figure 1A).

**Figure 2:**
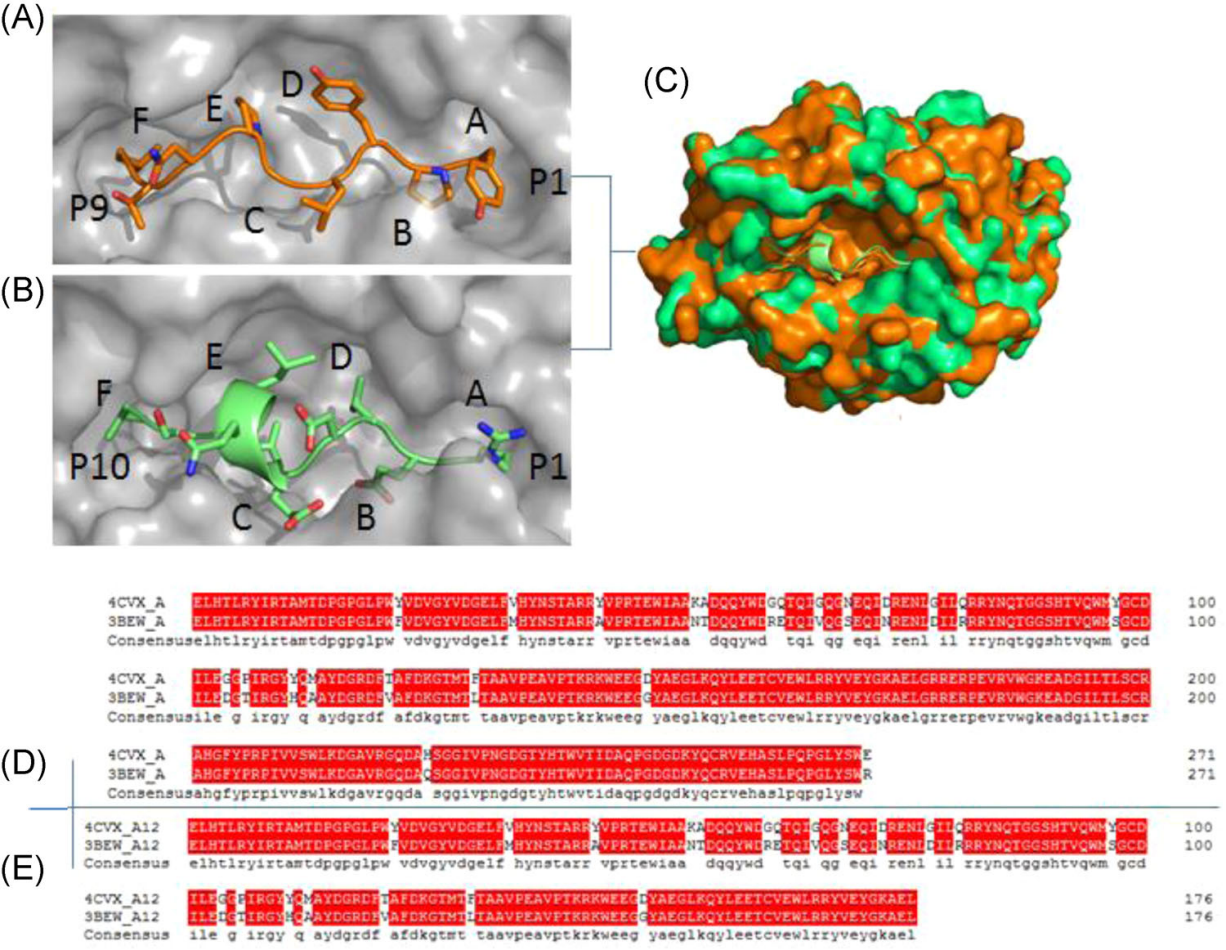
The peptide‐binding groove of BF2*0201and its comparison with BF2*2101. Comparison of the peptide‐binding grooves between BF2*0201 and BF2*2101, illuminating a relatively large binding groove of BF2*2101 and an extremely narrow groove of BF2*0201. (A, B) Molecular surfaces (grey) of the peptide‐binding grooves of BF2*0201 (A), BF2*2101 (B) with the peptides (YL9, RV10 colored in orange and green, respectively). The N‐(P1) and C‐(P9) termini of the peptides are marked. Pockets in each groove are sequentially labeled (A) to (F). (C) The surface of the BF2*0201‐YL9 binding groove is superimposed onto that of BF2*0401‐RV10. BF2*0201‐YL9 is shown in orange, while BF2*2101‐RV10 is in green. (D, E) Structure‐based amino acid sequence alignment of the α1–α2 domains of BF2*0201 and BF2*2101, with the secondary structure elements indicated above. Conserved residues are highlighted in red

Overlapping displays that the 4cvx‐YL9 structure closely agrees with the 3bew‐RV10 (Figure 1B), with the root‐mean‐square deviation between positions of the carbon backbone of A chain 0.558 Å (Figure 1C), of α1/α2 0.659 Å (Figure 1D), and of the B chain 0.337 Å (Figure 1E), and a same orientation of the β2m. However, there is a critical difference in the sequences of amino acids of 3bew and 4cvx (Figure 2D, E), and there is some difference in the DSSP of 3bew and 4cvx. Moreover, there is a visible difference in the ratio of the carbon backbone between them (Figure 1C).

### 3.2 By contrast BF2*0201, BF2*2101 has a relatively broad binding groove

The peptide binding grooves of 3bew and 4cvx are contrasted externally with one another under the same magnification. The 4cvx covered most of the 3bew, with three Pockets C, D, and E covering the main positions, while 3bew covered a few of the 4cvx, with two Pockets A and F covering the minor positions. Both 4cvx and 3bew have distinct pockets named A–F (Figure 2A–C).

Pocket A of 3bew and 4cvx is of exact identity. The properties of Pockets B, C, D, E, and F have differences in amino acid residues type as well as the number, especially Pocket F of the 3bew has one more hole than that of the 4cvx, and residues of Asn76, Tyr84, Gln112, Met113 Thr121, Ala122, Phe123, Phe130, and Lys141 were found in the 3bew peptide‐binding groove, while residues of Ala113, Ala114, Tyr115, Val121, and Leu130 in the 4cvx.

It is the difference of conformation caused by these diverse amino acid residues that made the binding groove size of 4cvx (1330 Å³) and 3bew (1570 Å³) difference by 240 Å³ in volume (Figure 2A–C). This shows a remarkable difference between the 3bew and 4cvx, and 3bew has a broader binding groove than that of 4cvx.

### 3.3 Contrast of the hydrophilic/hydrophobic properties and chargeability of the pockets of 4cvx and 3bew

The hydrophilic/hydrophobic properties and chargeability of the pockets of 4cvx and 3bew affect the sequence of peptide binding. We establish that for both 4cvx and 3bew, the hydrophobic character of Pockets A, B, D, E, and F is of dominance by analyzing the pockets of 4cvx and 3bew, yet the Pocket C of 4cvx was likewise hydrophobic and hydrophilic while that of 3bew primarily hydrophilic. As for chargeability, Pocket A of both is of identity uncharged, Asp24 of 4cvx as well as Asp24 and Glu62 of 3bew in Pocket B are negatively charged, and Asp73 of 4cvx in Pocket C is negatively charged while that of 3bew possess only a common positive charge of Arg9, and in Pocket D both 4cvx and 3bew possess only a public positive charge of Arg9, and in the Pocket E in addition to the public positive charge of Arg9 the 3bew has positive charges from His111, while there has only the public positive charge from Arg9 in that of 4cvx, and in the 4cvx the Pocket F has a positive charge from Arg83, Lys141, and Lys143, while there has Arg83 and Lys143 that are positively charged in that of 3bew. To make a long story short, the pockets of 4cvx and 3bew are generally hydrophobic and accord closely with the no sensitivity conservatism of BF2 that is more conservative to the outside world, which offers a good opportunity for binding peptides.

### 3.4 Specific amino acids make the central binding groove of 3bew highly broad

**Figure 3:**
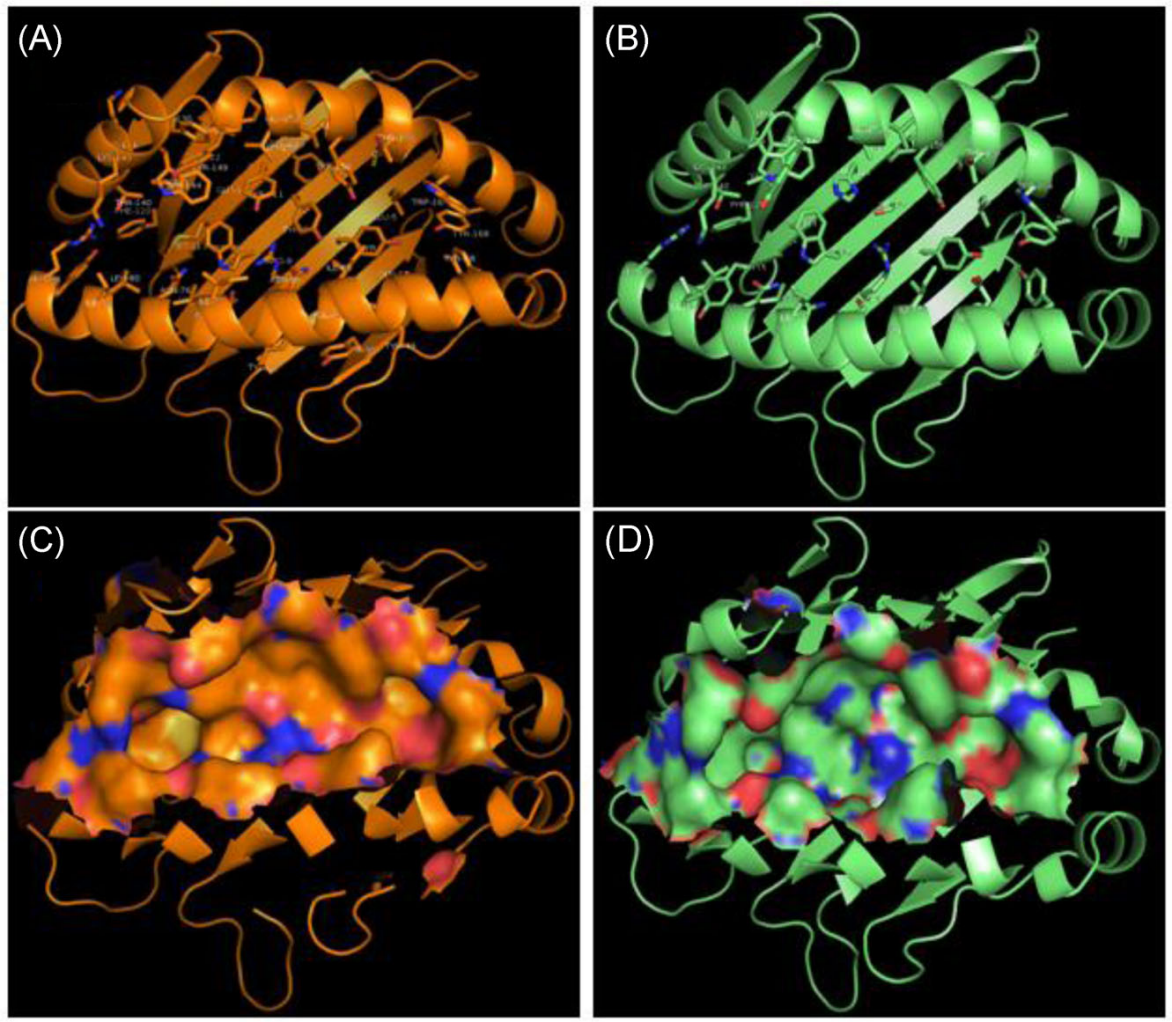
Peptide binding groove amino acid and the molecular surface of BF2*0201and BF2*2101. (A) Peptide binding groove amino acid of BF2*0201. (B) Peptide binding groove amino acid of BF2*2101. (C) Peptide binding groove molecular surface of BF2*0201. (D) Peptide binding groove molecular surface of BF2*2101

The conformations of the peptide‐binding groove are determined by their amino acid, especially the charged ones in the Pockets C, D, and E, bottom the amino acid of whom exert enormous influence on the binding groove. And the 3bew has the most common positively charged Arg9 there, with long flexible side chains and the negatively charged Asp73 and Asp24 (Figure 3A), while the 4cvx has the most common positively charged His111 and Arg9 there, with long flexible side chains, and the Asp24 at that of Pocket B (Figure 3B), and Glu62 at the center‐right of Pocket B.

Replacement of Tyr111 in 4cvx (Figure 3B) with His111 in 3bew (Figure 3A) makes the central binding groove of 3bew broader than that of 4cvx (Figure 3C,D) and that can be further compounded by that binding groove that comprised only 7 tyrosine residues (7 of 39) in 3bew while 12 (12 of 43) in 4cvx.

### 3.5 Contrast of the correspondence between residues of peptides and pockets of 4cvx and 3bew

**Figure 4:**
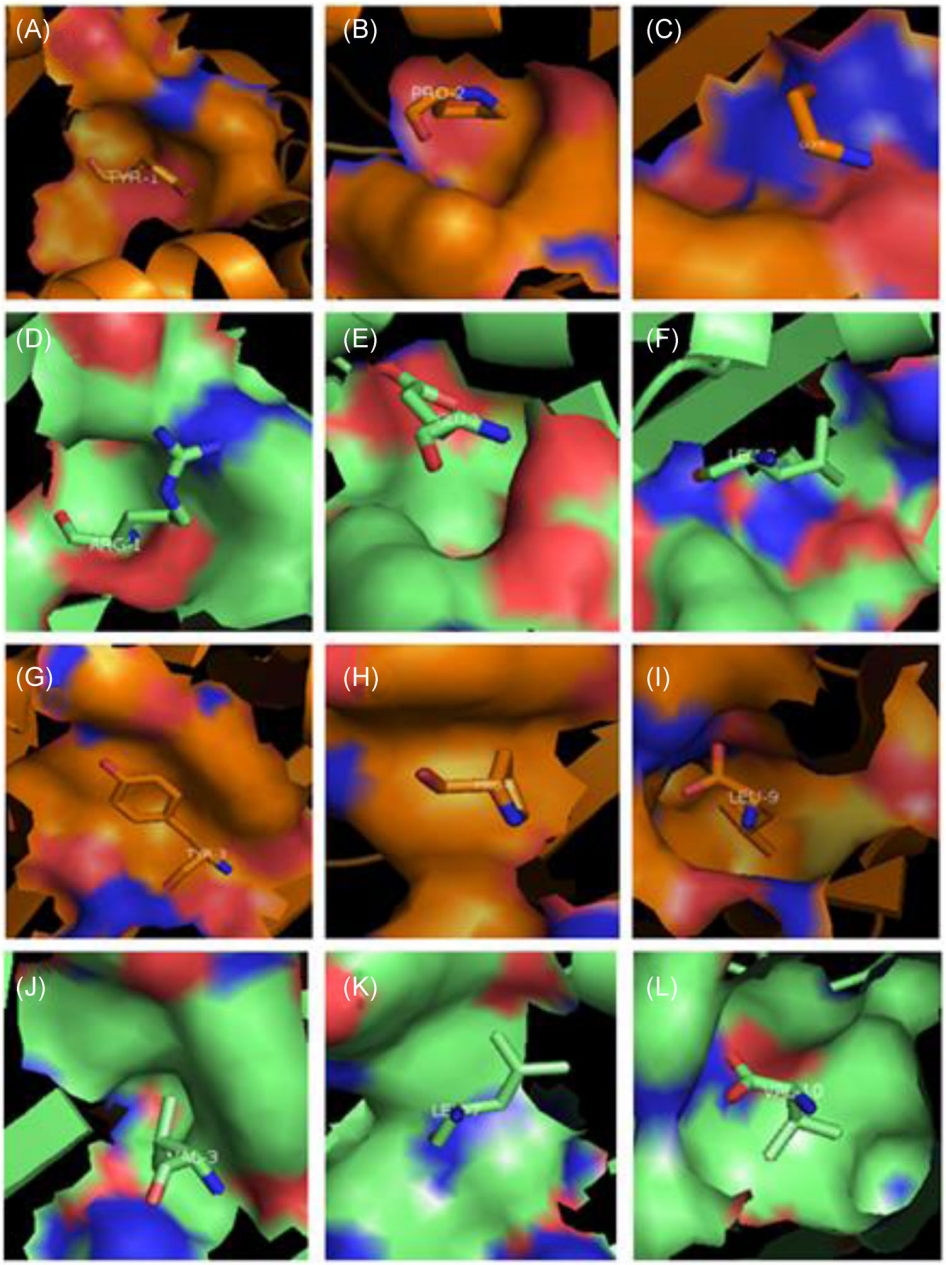
Corresponding relationships of pockets and amino acids in BF2*0201andBF2*2101, respectively. (A) The Pocket A and corresponding amino acids of BF2*0201. (B) The Pocket B and corresponding amino acids of BF2*0201. (C) The Pocket C and corresponding amino acids of BF2*0201. (D) The Pocket A and corresponding amino acids of BF2*2101. (E) The Pocket B and corresponding amino acids of BF2*2101. (F) The Pocket C and corresponding amino acids of BF2*2101. (G) The Pocket D and corresponding amino acids of BF2*0201. (H) The Pocket E and corresponding amino acids of BF2*0201. (I) The Pocket F and corresponding amino acids of BF2*0201. (J) The Pocket D and corresponding amino acids of BF2*2101. (K) The Pocket E and corresponding amino acids of BF2*2101. (L) The Pocket F and corresponding amino acids of BF2*2101

The presenting antigenic peptides of 4cvx and 3bew are 9 and 10 mer peptides, respectively, and the homologs of the pockets are different: they are P1 Arg‐Pocket A, P2 Glu‐Pocket B, P8 Leu‐Pocket C, P3 Val‐Pocket D, P7 Leu‐Pocket E, and P10 Val‐Pocket F, respectively, for 3bew (Figure 4A‐C,G–I); while they are P1 Tyr‐Pocket A, P2 Pro‐Pocket B, P8 Leu‐Pocket C, P3 Val‐Pocket D, P6 Pro‐Pocket E, and P9 Leu‐Pocket F, respectively, for 4cvx (Figures 4D–F,J–L). The homologous amino acid positions of the pockets of 4cvx and 3bew are similar.

### 3.6 Contrast of the BF‐peptide interaction of 4cvx and 3bew

**Figure 5:**
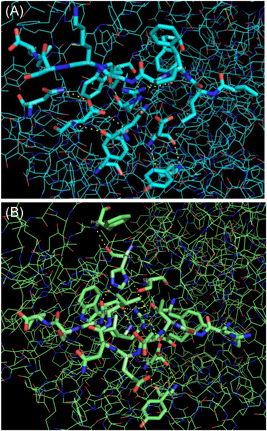
Hydrogen bonds of three important specific amino acids of BF2*0201 and BF2*2101, respectively. (A) The Arg9, Asp24, and Asp73 of BF2*0201 make the groove weak negative charge and, to some extent, tend to combine with the positively charged peptide. (B) The Arg9, Asp24, and His111 of BF2*2101 make the groove positive charge and extremely tend to combine with the negatively charged peptide, but the large space in the middle can also accommodate other amino acids

In 4cvx, nine hydrogen bonds and one salt bond were formed between binding groove and peptide, while fourteen and one in 3bew, respectively. More hydrogen bonds made the binding more firm in 3bew than in 4cvx. The Arg9 of 3bew has, respectively, a hydrogen bond and salt bond with peptide P2Glu, and Ser97 a hydrogen bond with peptide Tyr7, and Asp24 two hydrogen bonds with Tyr36, and His111 a hydrogen bond with P7Leu, Ser97, and Phe123, respectively (Figure 5A). Moreover, due to the specific amino acids‐Arg9, Asp24, and Asp73 of 4cvx and Arg9, Asp24, and His111 of 3bew, the effect of the polypeptide and the binding groove differs between the two and 3bew tends to bind polypeptides with negatively charged amino acids, but the large space in the middle can also accommodate other amino acids.

## 4 DISCUSSION

Resistance to lymphomas originating from pathogenic microorganisms,^8^ such as MDV and/or RSV^9^ and/or avian influenza viruses,^10^ has a close connection with the BF in the chicken.

The BF2 gene has achieved a dominant position in expressed BF gene, postulating as answerable for the MHC‐related Marek's disease (MD) and/or MHC‐related Rous sarcoma (RS) resistance. This study provides at least one point to elucidate the interaction of MHC–peptide that may inspire and fire the imagination through understanding the problems of MHC‐related MD and/or MHC‐related RS: Pockets C, D, and E play an important role in the conformations of the peptide‐binding groove, notably bottom the amino acid of whom exert a great impact on the binding groove. With long flexible side chains and the negatively charged Asp73 and Asp24, 3bew has the most common positively charged Arg9 there, while the 4cvx has the most common positively charged His111 and Arg9 there, with long flexible side chains, and the Asp24 at that of Pocket B, and Glu62 at the center‐right of Pocket B. Substituting His111 of 3bew Tyr111 of 4cvx further makes the central binding groove in 3bew broader than that of 4cvx. This result is consistent with the findings of Koch et al.,^7^ and their work shows that the binding groove has a remarkably great central cavity that affords extensive structure flexibility to the important residue Arg9, affording varying of crucial peptide‐binding sites. What's more, their work shows,^7^ by using a charge‐transfer system unheard‐of in BF, that the coupled variation of anchor residues affords peptides with notably diverse sequences to be bound, and their work shows^7^ that the anchor residue motifs of B21 are as follows: x‐(H, K or R)‐x‐x‐x‐x‐x‐x‐(E or D)‐x‐(A, V, L, I, F or W); x‐(H, K or R)‐x‐x‐x‐x‐x‐(E or D)‐x‐(A,V, L,I, F or W); x‐(E or D)‐x‐x‐x‐x‐x‐L‐x‐(A, V, L, I, F or W); x‐(E or D)‐x‐x‐x‐x‐x‐x‐L‐x‐(A,V, L, I, F or W), while B4: x‐(D or E)‐x‐x‐(D or E)‐x‐x‐E; B12: x‐x‐x‐x‐(V or I)‐x‐x‐V; B15: (K or R)‐R‐x‐x‐x‐x‐x‐Y and (K or R)‐R‐x‐x‐x‐x‐x‐x‐Y. But anchor residue motifs of B have not been studied yet. This result is basically coincident with the findings of Vishwa et al.^9^ (B21 has less mortality and was resistant to RSV‐A tumor, while B15 susceptibility to RSV‐A tumor) and Hunt et al.^10^ (B21 has less mortality and was resistant to H5N1 viruses). They concluded that this miscellaneous binding furthers our grasp of modes that BF can present peptides to the T lymphocytes and may account for the resistance of the B21 haplotype to MD.

The research is dedicated to the 3D structure of 4cvx from the B2 haplotype and the contrast of the BF‐peptide interaction of 4cvx and 3bew from the B21 haplotype. The conclusion of this study agrees well with data given in the literature^7^: there are usually various kinds of peptides presented by the BF2*2101 molecules of B21 haplotypes, resulting in resistance to pathogenic microorganisms, such as RSV and/or MDV.

## CONFLICT OF INTERESTS

The authors declare that there are no conflict of interests.

## AUTHOR CONTRIBUTIONS

All authors were involved in drafting the article or revising it critically for important intellectual content, and all authors approved the final version to be published. Dr. Jin had full access to all of the data in the study and takes responsibility for the integrity of the data and the accuracy of the data analysis. Study conception and design: Yuan‐chang Jin, Wei Wang, Min‐min Yu, Mei‐lin Hao, Gang Zeng, Jing‐fen Chen, Juan Dai, Yu‐jie Wu. Acquisition of data: Wei Wang. Analysis and interpretation of data: Yuan‐chang Jin, Wei Wang, Min‐min Yu, Mei‐lin Hao, Gang Zeng, Jing‐fen Chen, Juan Dai, Yu‐jie Wu.

## Data Availability

Due to the nature of this study, participants of this study did not agree for their data to be shared publicly, so supporting data is not available.
